# Beyond the Expiratory Limb: A Complete Raw Spirometry Dataset

**DOI:** 10.3389/fphys.2022.898831

**Published:** 2022-05-23

**Authors:** Daniel L. Ibraheem, Bishoy Samy, Jennifer H. Therkorn, Michael J. Falvo

**Affiliations:** ^1^ New Jersey Institute of Technology, Newark, NJ, United States; ^2^ Airborne Hazards and Burn Pits Center of Excellence, War Related Illness and Injury Study Center, VA New Jersey Health Care System, East Orange, NJ, United States; ^3^ Department of Pharmacology, Physiology and Neuroscience, New Jersey Medical School, Rutgers the State University of New Jersey, Newark, NJ, United States; ^4^ Department of Physical Medicine and Rehabilitation, New Jersey Medical School, Rutgers—The State University of New Jersey, Newark, NJ, United States

**Keywords:** spirometry, maximal expiratory flow volume, respiratory function test, data analysis, pulmonary ventilation

## Introduction

Kouri and colleagues (Kouri et al., 2021) recently described the 175-years history of spirometry, highlighting notable advances in our understanding of the respiratory system as well as technology for evaluation. One such example included Fry and Hyatt’s description of the *flow-volume curve* (Hyatt and Black, 1973) that advanced pulmonary physiology, yet since its introduction has largely focused on expiratory flow without much consideration to inspiratory flow. A lack of consideration to inspiratory flow during spirometry maneuvers has persisted for many years leading some to editorialize this oversight as “the neglected child of pulmonary diagnostics” (Ruppel, 2009). In addition to diagnostics, inattention to the inspiratory limb of the flow-volume curve had also been overlooked with respect to spirometry performance and acceptability criteria (Haynes and Kaminsky, 2015).

The most recent revision to the American Thoracic and European Respiratory Societies (ATS/ERS) technical standards on spirometry, however, has changed course and underscored the importance of coaching a full inspiration before and after a forced expiration with great vigor (Graham et al., 2019). Moreover, assessment of forced *inspiratory* vital capacity is now one of the acceptability criteria needed for a valid effort. Given new attention to the inspiratory portion of the flow-volume curve, there is an apparent need to characterize and translate this new data into clinically meaningful applications.

With the recent rise in emphasis of the inspiratory maneuver come several findings on important clinical applications of inspiratory based parameters. This increase in application of inspiratory measures suggests room for more studies regarding these measures. Peak inspiratory flow has proven to be an effective indicator of the success of specific bronchodilators in cases of asthma and other illnesses (Mahler, 2017). The forced inspiratory flow at 50% (FIF50), used in conjunction with its forced expiratory equivalent (FEF50) has been important as an indicator of upper airway obstruction (ATS/ERS 2005 criteria). However, some of these inspiratory indices are limited by poor reliability and high variability, as we can see with FEF50/FIF50 (García-Pachón et al., 1994). This can potentially contribute to the lack of consideration for these parameters, thus reducing awareness of the general benefits of studying the inspiratory limb, which is why the study of new parameters in this limb can be promising.

We have previously presented an abstract to describe an alternative index to quantify the inspiratory loop (Ibraheem et al., 2021), which served as the motivation for this data report. The calculation of the area of the inspiratory portion (AIN) offers a quantitative measure of the general shape of the flow-volume curve, comparable to similar findings of the expiratory portion found by Vermaak and colleagues (Vermaak et al., 1979) Measures such as these, as well as new possible parameters that can be found in the future, would need raw spirometry datasets that include the inspiratory portion as well as the expiratory portion. The National Health and Nutrition Examination Survey dataset, the largest and most widely used publicly available dataset, provides raw curve data for only the expiration, while the inspiration is not included (Centers for Disease Control and Prevention CDC, 2012). In this dataset, we hope to offer raw data of both the expiratory and inspiratory limbs that will be useful for further research into clinical applications.

## Methods

### Sample Population

Spirometry data were obtained from 130 healthy adults participating in a repeated-measures study designed to assess intra- and inter-session variability, details of which have previously been reported (Therkorn et al., 2021). In brief, healthy non-smoking adults between the ages of 18–40 years old completed spirometry on two separate laboratory visits separated by 7 days (±3 days) and performed at the same time of day (±2 h). Study procedures were approved by the Rutgers University Institutional Review Board and all participants provided their written informed consent.

### Data Acquisition

A single-trained technician attempted to collect a minimum of 8 spirometry maneuvers or trials for each participant at each visit. After quiet tidal breathing on the spirometer, participants were coached to perform a maximal inhalation followed by a maximal exhalation to residual volume (i.e., plateau), followed by a second maximal inhalation. The spirometer (Easy-on PC spirometer; nDD Technologies, Zurich, Switzerland) utilized ultrasonic transit-time measurements with a sampling frequency of 400 Hz and accuracy of ±2% for volume and flow. A calibration check with a 3-L syringe was performed prior to each participant’s test session. Technician’s instructions and coaching cues were standardized, and participants were provided approximately 60 s of rest between trials. Trials were performed in a seated position and the participant donned a noseclip and made a tight seal around the breathing tube (ndd spirette™). Additional details regarding data acquisition are described elsewhere (Therkorn et al., 2021).

### Data Reduction

Offline review of all trials was performed by a single experienced investigator using the manufacturer’s software (EasyOne Connect; nDD Technologies) in accordance with the American Thoracic and European Respiratory Societies (ATS/ERS) guidelines for acceptability (Graham et al., 2019). Unacceptable trials were manually flagged within the software. Raw data (XML format) were exported from the software and converted to a comma-separated values file. Raw flow and volume data for each patient was then imported into MATLAB for additional analyses. A time series was created based on the 100 Hz sampling frequency for each accepted trial of each subject.

## Dataset Description

### Descriptive Characteristics

The dataset [10.6084/m9.figshare.19196543] is comprised of 2,287,213 observations (1,055,236 observations after removing rows labeled as “NaN” to indicate “not a number”) and 6 variable columns where each observation is unique to a given subject, visit, trial and pair of time series measurement values for flow and volume. The dataset is provided in long format but can be easily pivoted to wide format if that is desired. This is the reasoning for including NaN rows to pad each trial to match the same length of the longest subject’s trial; some software may be easier to pivot the dataset with this padding included while others may prefer to strip these NaN rows. There are a total of 129 subjects present in this dataset as some were unable to achieve complete flow volume loop data with an inspiratory portion (n = 129/137, 94.2% with complete data). The six variables are specifically defined in Table 1. While Table 1 summarizes the dataset as a whole, at the subject level the mean number of trials was 8.2 ± 1.8 trials per subject (across both visits). There are a total of 1972 different trials present in the dataset with 1,004 of these from first test sessions (1,004/1972, 50.9%) and 968 of these from second test sessions (968/1972, 49.1%). A complementary demographics dataset is also provided with one observation per subject (129 rows of data) and demographic measurements as summarized in Table 2 below.

**TABLE 1:** Main dataset variables summary.

Variable	Definition	Values Range
ID	Uniquely assigned subject ID number	101–237
VISIT	Study session number	1–2
TRIAL	Sequentially numbered trial for each subject and visit	1–16
TIME	Time stamp of flow and volume measurement (ms)	0–10,750
VOLUME	Volume measurement corresponding to flow volume loop (L)	−0.405–6.409
FLOW	Flow measurement corresponding to flow volume loop (L/s)	−8.88690–14.24400

**TABLE 2:** Demographics dataset variables summary.

Variable	Definition	Values Range
ID	Uniquely assigned subject ID number	101–237
WEIGHT	Subject weight (kg)	43.00–143.00
HEIGHT	Subject height (cm)	151.0–193.0
SEX	Subject birth sex	39 Male, 90 Female
AGE	Subject age on date of study visit (yr)	18–39
ETHNICITY	Subject reported ethnicity	19 Hispanic or Latino, 108 Not Hispanic or Latino, 2 Missing Data
RACE	Subject reported race(s)	37 Asian, 20 Black or African American, 58 White, 7 > 1 Race, 7 Missing Data

### Application Example

Our script (Supplementary Material S1) focused on the inspiratory portion of the flow-volume loop and calculating an index referred to as the area under the inspiratory curve (AIN) (Supplementary Figure S1). Certain criteria were developed to ensure a best possible area. This criterion includes ensuring the negative flow, or inspiration, is greater than 1 L/s to filter out any data which may not seem to be maximal effort. Inspiratory volume must also have reached within 0.5 L of total lung capacity to be accepted for AIN calculations. AIN was calculated using trapezoidal numerical integration applied to the inspiratory curve. The outputs of this script come as a figure that displays the AIN on the flow-volume curve of each trial (Supplementary Figure S2).

The calculation of AIN required the raw data because the entire flow-volume loop was needed to reconstruct the graph and calculate this area. This particular parameter shows some promise, possibly due to its unique approach of quantifying the entire shape of the inspiratory loop instead of viewing a measurement at a particular point in time. New approaches to analyzing the inspiratory limb, such as AIN, would require access to a database containing raw measurements.

## Conclusion

We provide a raw spirometry dataset, including both inspiratory and expiratory curve data, obtained from 129 healthy adults who volunteered to participate in a research study (Therkorn et al., 2021). Each individual performed multiple forced vital capacity maneuvers in accordance with a rigorous and standardized protocol on two separate occasions. All maneuvers were collected by a single trained technician and quality checked in accordance with the current ATS/ERS guidelines (Graham et al., 2019). Raw data were exported directly from the manufacturer’s software (csv format) and modified to adhere to tidy data standards. To our knowledge, publicly available spirometry datasets are presently limited to expiratory data only [.e., National Health and Nutrition Examination Survey ((Centers for Disease Control and Prevention (CDC)))]; therefore, the present dataset may foster additional research directed at the inspiratory portion of forced vital capacity maneuvers as well as encourage manufacturer’s to consider providing additional variables specific to the inspiratory portion of spirometry.

## Data Availability

The datasets presented in this study can be found in online repositories. The names of the repository/repositories and accession number(s) can be found below: https://doi.org/10.6084/m9.figshare.19196543.v1.
