# Supplementary material for: Beyond the Expiratory Limb: A Complete Raw Spirometry Dataset
Source: Front Physiol. 2022 May 23;13:898831. doi: 10.3389/fphys.2022.898831 (PMC9168312; doi:10.3389/fphys.2022.898831)
Supplement: Supplementary file 1 [file DataSheet1.docx]

Supplementary Material

# Supplementary Data

To illustrate how raw inspiratory spirometry data may be utilized, we present a practical example of how to calculate the area above the inspiratory flow-volume curve, or what we refer to as AIN (Figure S1). We calculated AIN using a custom written MATLAB script on the dataset accompanying this article (10.6084/m9.figshare.19196543). This code is described below and provides an example of how the dataset can be used to explore new indices in inspiratory maneuvers using this complete and raw dataset.

Importing Data

The data are imported from a text file of choice into MATLAB. The file must be structured as a tidy data set, with column titles of *Subject ID, Visit Number, Trial Number, Time, Volume, Flow*. Our dataset is structured in this way and works effectively in this script.

In this first part, the user is given the opportunity to choose the file that will be analyzed using a dialog box.

clear; clc; clf; close all

% Script for importing data from spreadsheet

set(0, 'DefaulttextInterpreter', 'none') %change text interpreter so no longer creates subscripts in titles

filterspec ={'*.xlsx;*.xls'};

original_path = pwd;

Title = 'Select text file to analyze:';

[filename, pathname] = uigetfile('*.txt', Title, 'MultiSelect', 'on');

if isequal(filename,0) || isequal(pathname,0)

disp('User pressed cancel')

else

disp(['User selected: ', fullfile(pathname, filename)])

end

cd(pathname);

current_directory = cd;

Here, the selected file is imported into MATLAB and converted to a form that will be helpful when restructuring the data.

%% Import data from text file

% Setup the Import Options and import the data

opts = delimitedTextImportOptions("NumVariables", 6);

% Specify range and delimiter

opts.DataLines = [1, Inf];

opts.Delimiter = ",";

% Specify column names and types

opts.VariableNames = ["VarName1", "VarName2", "VarName3", "VarName4", "VarName5", "VarName6"];

opts.VariableTypes = ["double", "double", "double", "double", "double", "double"];

% Specify file level properties

opts.ExtraColumnsRule = "ignore";

opts.EmptyLineRule = "read";

% Import the data

tidydata = readtable(filename, opts);

tidydata = table2array(tidydata);

%% Clear temporary variables

clear opts

Restructuring the data

Several variables are saved from the dataset so that they can be used for the calculation of AIN and the organization of the output data. This is done through a loop that goes through each subject and each trial.

%% Create data variables

%subject IDs

subID=unique(tidydata(:,1)); %Pulling IDs from first column

all_patients=subID;

num_patients=length(all_patients);

%% Preparing data to run AIN calculation on each individual trial for each patient

for i = 1:num_patients;

ID=all_patients(i);

%Creating folder for patient

%saves name of directory of subject

sub_directory=strcat(current_directory,'\ID',num2str(ID));

%creates folder for subject

mkdir(sub_directory);

cd(sub_directory);

%Finding all rows with ID number

index_ID=find(tidydata(:,1)==all_patients(i));

%Assigning new variable name for all individual patient data

patientdata=tidydata(index_ID,:);

%Separating data by visit (located in column 2)

visit_id=tidydata(index_ID,2);

%Initializing desired data

accepted_flow=[];

accepted_volume=[];

trials_array=[];

visit=[];

%Identifying number of visits

tot_visit=unique(visit_id);

%Loop by visit

for j = 1:length(tot_visit);

index_visit=find(patientdata(:,2)==tot_visit(j));

visitdata=patientdata(index_visit,:);

%Identifying trials (located in column 3)

trials=unique(visitdata(:,3));

%Identifying volume and flow within a loop to create

%matrix of flow and volume for all of the subject's trials

for k = 1:length(trials);

% Identifying each trial

trial_curr=trials(k);

visit=[visit tot_visit(j)];

trials_array=[trials_array trial_curr];

index_trial=find(visitdata(:,3)==trials(k));

%creating volume and flow

time_trex=visitdata(index_trial,4);

vol_trex=visitdata(index_trial,5);

flow_trex=visitdata(index_trial,6);

accepted_flow=[accepted_flow flow_trex];

accepted_volume=[accepted_volume vol_trex];

end

end

%Transpose Array

accepted_flow=accepted_flow';

accepted_volume=accepted_volume';

accepted_trials=length(trials_array);

%dimension for subplots in future

subplot_size=ceil(sqrt(length(trials_array)));

m=0;

**AIN Calculation**

In this section, the AIN is calculated by finding the integral using the trapezoidal rule. The script is designed to include only negative data and exclude the expiratory portion and any tidal breathing. There are two major criterion that deem a trial acceptable or unacceptable for AIN calculation. The first is that the inspiratory curve must return to at least -0.5 L/s so that it is close enough to the x-axis and can be considered a complete maneuver. The second is that the inspiratory flow must reach a peak of at least 1 L/s so that it can be considered a sufficient inspiratory effort. Trials that do not meet this criterion are outputted as having an AIN value of 0.

%% AIN Calculation

vol_flow_graph_ain=figure;

%Initializing AIN

AIN=[];

%Looping through trials to calculate AIN

for n =1:accepted_trials;

%Using volume and flow for specific trial

vol_ain=accepted_volume(n,:);

flow_ain=accepted_flow(n,:);

%Counting number of times looped for future subplot

m=m+1;

% Find 2nd idx for ain calc

%Finding peak of inspiratory (since inspiratory is negative

%flow, it will be the minimum value)

[min_flow min_flow_idx] = min(flow_ain);

%Max volume represents begining of inspiratory manuever

[max_vol max_vol_idx] = max(vol_ain);

[ain_val2 ain_val2_idx]=find(flow_ain(min_flow_idx:end)>=0);

%Acceptance Criteria: Inspiratory Flow must be greater than 1

%L/s and Maximum volume must be greater than 0.5 L in order to

%proceed with AIN calculation

if abs(min_flow)>1 && max_vol>0.5;

%Ensuring inspiratory curve is where data first enters

%negative flow up until it first passes into positive flow

if isempty(ain_val2) && isempty(ain_val2_idx);

%Acceptance Criteria: inspiratory flow must be as close

%as 0.5 L/s away from 0 to proceed with calculation

if min(abs((flow_ain(min_flow_idx:end))))>0.5;

ain_val2=nan;

ain_val2_idx=nan;

disq=1;

else

[ain_val2 ain_val2_idx]=find(isnan(flow_ain(min_flow_idx:end)));

ain_val2=ain_val2(1);

ain_val2_idx=ain_val2_idx(1);

% ain_end_idx=length(neg_flow_ain);

end

else

ain_val2=ain_val2(1);

ain_val2_idx=ain_val2_idx(1);

end

%End index of whole data

ain_idx2=ain_val2_idx+min_flow_idx-2;

% Find first index for calculating ain - Second Part

%We flip flow in order to progress from the minimum to where flow

%crosses 0 into the positive.

flip_flow_ain=flip(flow_ain);

min_flow_idx_flip=length(flow_ain)-min_flow_idx+1;

[ain_val1_flip ain_val1_idx_flip]=find(flip_flow_ain(min_flow_idx_flip:end)>0);

ain_val1_flip=ain_val1_flip(1);

ain_val1_idx_flip=ain_val1_idx_flip(1)-1;

ain_idx1=length(flow_ain)-(ain_val1_idx_flip+min_flow_idx_flip)+2;

else

ain_idx2=nan;

ain_idx1=nan;

end

%If either of acceptability criteria was not met, disqualify

%trial

if exist('disq');

ain_idx2=nan;

ain_idx1=nan;

end

figure(vol_flow_graph_ain);

%If data is disqualified, AIN is set to 0 and flow-volume curve is plotted for visual analysis by user

if isnan(ain_idx1) && isnan(ain_idx2);

neg_vol_ain=vol_ain;

neg_flow_ain=flow_ain;

AIN(n)=0;

subplot(subplot_size,subplot_size,m);

plot(neg_vol_ain,neg_flow_ain,'r'); hold on;

xlabel(strcat('Trial',num2str(trials_array(m)),' AIN: ',num2str(AIN(n))));

else

%Area is computed by integration of inspiratory limb using trapezoidal method

neg_vol_ain=vol_ain(ain_idx1:ain_idx2);

neg_flow_ain=flow_ain(ain_idx1:ain_idx2);

AIN(n)=trapz(neg_vol_ain,neg_flow_ain);

%Plotting all trials for each subject

subplot(subplot_size,subplot_size,m);

plot(neg_vol_ain,neg_flow_ain,'r');

hold on;

plot(vol_ain(ain_idx1:ain_idx2),flow_ain(ain_idx1:ain_idx2),'bo'); hold on;

area(vol_ain(ain_idx1:ain_idx2),flow_ain(ain_idx1:ain_idx2));

xlabel(strcat('Visit',num2str(visit(m)),'Trial',num2str(trials_array(m)),' AIN: ',num2str(AIN(n))));

end

clear disq;

end

**Exporting Data**

The resulting AIN calculations are saved as images showing the AIN as figures or images of a subplot containing all the analyzed trials, images of the inspiratory curves, and the AIN values. It then outputs the data into an excel sheet containing the AIN values organized by ID, visit, and trial. The data is presented in the format of several subplots as shown in Figure S2.

sgtitle(strcat('AIN ID: ',num2str(ID)));

saveas(vol_flow_graph_ain, ID+"AIN.jpg")

saveas(vol_flow_graph_ain, ID+"AIN.fig")

close(vol_flow_graph_ain);

save(strcat(num2str(ID)));

%end of AIN

end

# Supplementary Figures and Tables

## Supplementary Figures

##
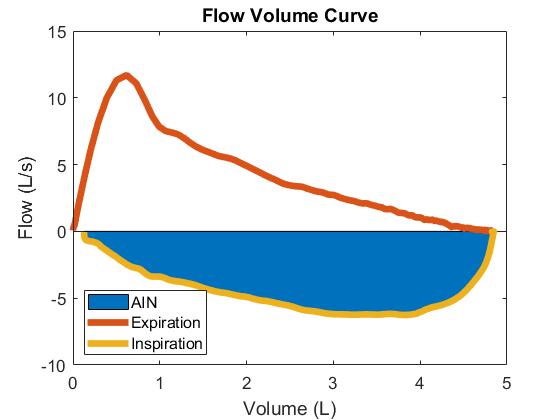


## Supplementary Figure 1. The graph above uses an example to give a visual display of AIN, shown as the area below the x-axis and above the inspiratory curve. It is shown in the area shaded in blue.


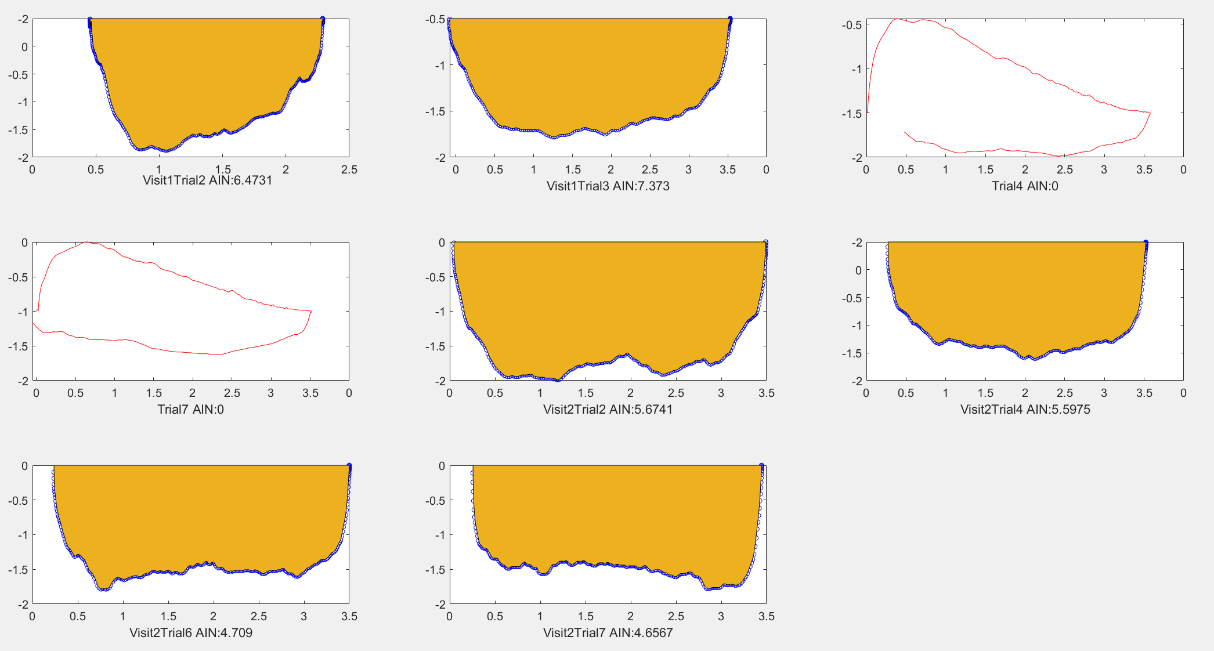


**Supplementary Figure 2.** Above is an example of the output of an AIN calculation produced by the MATLAB script above. Accepted maneuvers are shown by shaded orange inspiratory curves. Any unacceptable trials results in an AIN of 0 and graphs the complete flow volume curve. The AIN is shown below the graph.
